# Predicting changing malaria risk after expanded insecticide-treated net coverage in Africa

**DOI:** 10.1016/j.pt.2009.08.002

**Published:** 2009-11

**Authors:** David L. Smith, Simon I. Hay, Abdisalan M. Noor, Robert W. Snow

**Affiliations:** 1 Department of Biology and Emerging Pathogens Institute, University of Florida, P.O. Box 100009, Gainesville, Florida 32610, USA; 2 Spatial Epidemiology and Ecology Group, Tinbergen Building, Department of Zoology, University of Oxford, South Parks Road, Oxford OX1 3PS, United Kingdom; 3 Malaria Public Health and Epidemiology Group, Centre for Geographic Medicine, KEMRI – University of Oxford – Wellcome Trust Collaborative Programme, Kenyatta National Hospital Grounds (behind NASCOP), P.O. Box 43640-00100, Nairobi, Kenya; 4 Centre for Tropical Medicine, Nuffield Department of Clinical Medicine, University of Oxford, CCVTM, Oxford OX3 7LJ, UK

## Abstract

The Roll Back Malaria (RBM) partnership has established goals for protecting vulnerable populations with locally appropriate vector control. In many places, these goals will be achieved by the mass distribution of insecticide treated bednets (ITNs). Mathematical models can forecast an ITN-driven realignment of malaria endemicity, defined by the *Plasmodium falciparum* parasite rate (*Pf*PR) in children, to predict *Pf*PR endpoints and appropriate program timelines for this change in Africa. The relative ease of measuring *Pf*PR and its widespread use make it particularly suitable for monitoring and evaluation. This theory provides a method for context-dependent evaluation of ITN programs and a basis for setting rational ITN coverage targets over the next decade.

## Strategic plans and likely timelines for malaria control

The Abuja Declaration and Plan of Action (2000) set targets of protecting 60% of pregnant women and children under five years’ old with insecticide-treated bednets (ITNs) by 2005 [1]. The Roll Back Malaria (RBM) strategic plan (2005) subsequently redefined these targets to 80% coverage by 2010 [2] and the recent Global Malaria Action Plan (2008) called for a rapid scale-up to achieve universal coverage with some form of vector control [3]. There has been fast, large-scale ITN deployment in some areas of Africa, but ITN use in many parts of the continent remains low [4]. In areas where high ITN coverage has been achieved, there are early reports that the epidemiology of malaria in these areas is in transition [5–12], but the theoretical basis for attributing these changes to ITNs remains poorly defined. Scaling-up ITN coverage across Africa remains a high priority, but there is also a need to learn from the rapid scale-up of ITN coverage and put that information to work as countries define strategic plans and set funding priorities for the next five years and beyond. This planning process would benefit from a quantitative and predictive approach that is based on direct measures of malaria and ITN usage, not just estimates of commodity distribution. Using mathematical models, it is possible to define rational expectations about ITN-driven changes in malaria in relation to actual ITN usage, the ITN coverage levels required to achieve national goals for malaria reduction, and the likely timelines for change.

## Finding a metric

A predictive theory for ITNs is ideally based on quantities that are commonly and easily measured. One effect of ITNs is to reduce the personal risk of clinical malaria, severe malaria, and malaria mortality for the individuals who use them [13,14]. Changes in disease burden are the outcomes of greatest interest, but they are also the most difficult to measure. Population-level benefits occur because ITNs also slow transmission by increasing mosquito death rates, delaying feeding, or diverting some bites onto non-human hosts [13,14]. High levels of ITN ownership and usage by all members of a community can therefore substantially reduce the vectorial capacity, reduce the size of the parasite reservoir [15,16], and protect people who do not own a net [17–19].

Several metrics have been developed over the past century to measure these population-level parasitological, entomological, and epidemiological aspects of malaria transmission [20]. Three potentially useful metrics are the *Plasmodium falciparum* parasite rate (*Pf*PR or malaria prevalence, the proportion of the population positive for malaria infection, which is usually measured by microscopy), entomological inoculation rate (*Pf*EIR, the expected number of infectious bites per unit of time) and basic reproductive number (*Pf*R_0_, the expected number of malaria cases that would arise from a single case after one parasite generation if there were no malaria immunity or malaria control). The *Pf*R_0_ would be an ideal metric to use for planning [21], but fewer than 50 direct estimates have been made [22–24]. The annual *Pf*EIR provides a direct measure of exposure to malaria, and been measured hundreds of times [25], but not sufficiently extensively nor in a standardized way that would provide a sound basis for planning. The *Pf*PR is frequently measured, and more than 17,000 geo-referenced estimates of *Pf*PR made since 1985 have been age-standardized and assembled into a database by the Malaria Atlas Project (MAP) [26–29].

We have, therefore, developed a theory and forecast ITN-driven changes in endemicity, defined by the *Pf*PR in children aged 2–10 years (Box 1). The *Pf*PR provides a direct estimate of the reservoir of asexual parasites, so reductions in *Pf*PR provide a direct estimate of the progress towards control and elimination of disease [20,30]. The relative ease of measuring *Pf*PR in children aged 2–10 years and its widespread measurement [29] make it particularly suitable for strategic planning, monitoring and evaluation (Box 1).

## Malaria transmission models and control

Malaria transmission models provide a basis for developing and refining a predictive theory based around the *Pf*PR. Starting with Ronald Ross [31,32], malaria transmission models established a quantitative basis for evaluating the complex quantitative relationships between *Pf*PR, *Pf*EIR and *Pf*R_0_. These earlier theoretical models have now been extended to include simple models of malaria immunity [33], superinfection [34], heterogeneous biting [35], various modes of malaria control [36,37], and complex individual-based computer simulations [38,39].

**Figure 1:**
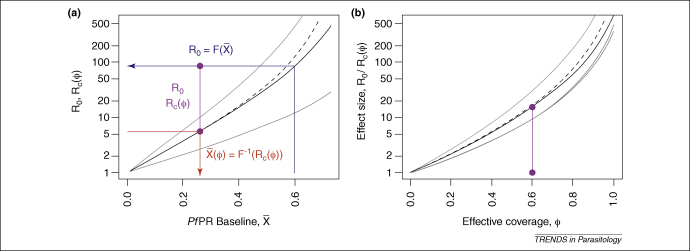
Predictive theory requires a transmission model integrated with a control model. (**a**) The malaria transmission model predicts a particular relationship between baseline *Pf*PR and *Pf*R_0_. The solid black line shows a population where 20% of the population gets 80% of the bites (α = 4.2); the dashed line shows the same degree of heterogeneous biting but with some immunity that blocks transmission to mosquitoes. The lower gray line shows the relationship in places where biting is more homogeneous biting (α = 2), implying lower *Pf*R_0_ for the same *Pf*PR) and the upper gray line shows the relationship in places where it is more heterogeneous (α = 6), implying higher *Pf*R_0_ for the same *Pf*PR). For example, the blue line suggests that *Pf*R_0_ is ∼85, starting from a baseline *Pf*PR of 60%. (**b**) The control model describes the proportional reduction in transmission as a function of effective coverage. The solid lines represent the bionomics of four vectors [14,45]. The dashed black line is the geometric mean for the two *An. gambiae* species from different places. The dark solid line is *An. arabiensis*, which was used as the benchmark. The purple segment shows the ITN effect size for 60% effective coverage, such as would occur with 80% ownership and 75% usage. To compute a new endpoint *Pf*PR, this reduction is used in part (a) to compute a new reproductive number under control, *Pf*R_C_(ϕ), and the new *Pf*PR endpoint, $\bar{X}\left(\phi \right)$ [see the purple segment and the red lines, in part (a)]. The same algorithm can be used to predict the change in *Pf*PR starting from one level of effective ITN coverage and switching to another.

Basic epidemiological theory for malaria suggests that *Pf*R_0_ defines a steady state for *Pf*PR [40], so a malaria transmission model and *Pf*PR can be used to estimate *Pf*R_0_ [24,41] (Figure 1a and Supplementary Online Information). Given the age-related patterns in *Pf*PR, it is necessary to use an age-standardized *Pf*PR to estimate the *Pf*R_0_; children aged 2–10 have poorly developed anti-parasite immunity but ample exposure to malaria, so *Pf*PR in these age groups best reflects the steady state [27].

To establish quantitative benchmarks for planning, a published malaria transmission model that describes superinfection, heterogeneous biting, and immunity was used; the model fits the empirically observed relationships between *Pf*EIR and *Pf*PR in African children better than a well-established statistical relationship [42,43], and the fitted parameters are consistent with direct observations [24,41]. The relationship between *Pf*PR, *Pf*EIR and *Pf*R_0_ is strongly affected by the degree of heterogeneous biting, which can disguise subpopulations with intense exposure. Contrast two populations with a *Pf*PR of 10%: in a population in which 10% of people are bitten many times each day, but in which 90% of the population is never bitten, *Pf*R_0_ would be much higher than in a population with a *Pf*PR of 10% with uniform biting rates. The relationship between *Pf*PR and *Pf*R_0_ from this model is shown graphically in Figure 1a.

A second model is required to model the effects of ITNs. A suitable model is based on the mosquito feeding cycle that describes changes in the vectorial capacity, the vector-related aspects of the reproductive number [44]. The effect of ITNs depends on the proportion of the whole community that owns and uses a net and the proportion of biting that occurs indoors at night, called the effective coverage (*ϕ*) [14]. Increased use of ITNs lowers the vectorial capacity, and reduces the reproductive number to a new level, *Pf*R_C_(*ϕ*). The ITN effect size on transmission, defined by the ratio *Pf*R_0_:*Pf*R_C_(*ϕ*), depends on effective coverage and vector bionomics. The predicted relationship between ITN effective coverage and the effect size for different vectors is illustrated graphically in Figure 1b.

Both models are necessary because of the non-linear functional relationships between *Pf*PR and *Pf*R_0_, and between ITN effective coverage and the transmission effect size. To compute a new steady state, the malaria transmission model uses the output of the ITN model. The same function that describes *Pf*R_0_ in terms of *Pf*PR is inverted to predict a new steady state for *Pf*PR in terms of *Pf*R_C_(*ϕ*) (Figure 1a).

**Figure 2:**
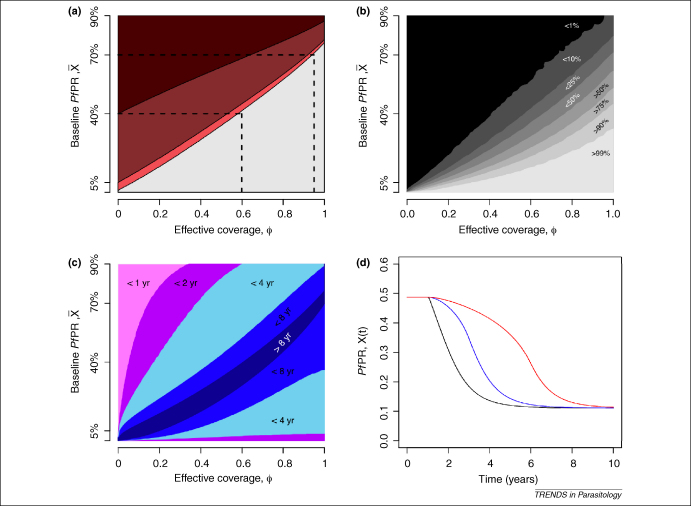
(**a**) For the benchmark parameters, the endemicity class of the *Pf*PR endpoint for every baseline *Pf*PR and every effective ITN coverage level (*ϕ*). The colors represent different endemicity levels (dark red, >40%; red, 5%–40%; pink, 1%–5%; and gray, <1%). The dashed black lines highlight two points, the level of effective coverage required to reduce *Pf*PR to below 1% starting from a baseline of 40% and a practical maximum starting point for which low stable endemic control is achievable with only ITNs, at 95% effective coverage. (**b**) The uncertainty associated with the benchmark prediction is represented here as the probability of reducing *Pf*PR to below 1%, given the uncertainty about biting heterogeneity and vector bionomics (Supplementary Online Information). (**c**) The changes in *Pf*PR do not happen instantaneously, even in the best case in which ITN coverage is rapidly scaled-up to the maximum and illustrated here. The colors show the waiting time until *Pf*PR is within 1% of the endpoint in Figure 2a (>8 years, dark-blue; 4–8 years, blue; 2–4 years, sky-blue; 1–2 years, purple; <1 year pink). When R_C_(ϕ) ≈ 1 so that the endpoint is approximately 1% (black region), the waiting times can be more than one decade [49]. (**d**) The timelines for changing *Pf*PR endemicity are sensitive to the rate that ITNs are scaled-up. These illustrate the changes over time starting from a baseline of approximately 50%, when the ITN coverage scales up to a maximum instantaneously (black), or linearly over a period of 2 years (blue), or 5 years (red). The relationship between ITN coverage and the effect size is greater than log–linear (see Figure 1b), so the maximum effect size is not achieved until ITN coverage levels are very close to the maximum value.

Benchmark predictions from these two models are based on the best-fit parameters from the malaria transmission model [41] and vector bionomics for a typical African vector (Figure 1b, Supplementary Online Information) [14,45]. The models predict the changes in *Pf*PR endpoints from any baseline and for any level of ITN effective coverage; the corresponding endemicity class of the endpoint is shown graphically in Figure 2a. The models suggest that the outcome of scaling up ITNs will vary, depending on baseline *Pf*PR, the ITN effect size, and the degree of heterogeneous biting (Figure 1a, and Supplementary Online Information). The ITN effect size varies with vector bionomics, the fraction of mosquitoes killed or repelled by the nets, and other factors [14]. The predictions are, thus, accompanied by an assessment of uncertainty (Figure 2b, and Supplementary Online Information).

## Setting targets

To be effective and transparent, country-level plans must set verifiable targets that are described as quantitative changes in malariometric indices. The theory developed here can provide guidance in setting these goals based on a commonly used metric. To illustrate how this can be done, two realistic benchmarks were set that have some utility for national malaria control programs when applying for international donor support: (i) what ITN coverage levels would be required to halve existing *Pf*PR? and (ii) what ITN coverage levels would be required to reach a national or sub-national goal of 1% *Pf*PR? At a 1% *Pf*PR, disease burdens across Africa would be substantially reduced [46–48].

If *Pf*PR is 70%, scaling-up ITNs to an effective coverage of 70% will ultimately halve this starting endemic level (Table 1). As a rule of thumb for halving *Pf*PR, the increase in effective coverage must be at least 80% of baseline *Pf*PR. What can be achieved with 80% ITN ownership used 75% of the time (i.e. 60% effective coverage), consistent with short-duration, but large-scale ITN trials [15,16]? At these levels, a reduction in transmission of 93% would reduce *Pf*PR to below 1% if the baseline *Pf*PR was below ∼40%.

ITNs do not provide perfect protection, so full coverage may not be sufficient to achieve sustained endemic control areas with very high baseline *Pf*PR. If the baseline *Pf*PR exceeds 70%, the models predict that 94% effective coverage is required to reach *Pf*PR of 1%. This would represent an upper limit in a context where 6% of biting by vectors occurred outdoors.

*Pf*PR does not change instantaneously. Timelines for changing malaria endemicity as ITN coverage is gradually scaled-up can be found by simulating malaria transmission in the corresponding models [49,50] (Supplementary Online Information). After reaching ITN coverage targets, the time to reach the new *Pf*PR endpoint can be as short as a few months. If the endpoint is stable endemic control, if *Pf*R_C_ (*ϕ*) is close to one, the waiting times can be more than a decade (Figure 2c)[49].

An important lesson was that timelines for ITN impact on *Pf*PR are extremely sensitive to the time taken to reach a scaled coverage target (Figure 2d). The predicted functional relationship between ITN effective coverage and proportional reductions in vectorial capacity is, in the model, greater than log–linear (see Figure 1b). The greatest reductions in vectorial capacity are realized when ITN coverage levels reach the target, usually near the end of the scaling-up period. National sample surveys should therefore compare *Pf*PR endpoints in a standard fashion and cross-sectional surveys be repeated for 3–5 years after ITN coverage reaches its target maximum.

Most African governments set strategies for malaria control, policy and financing on five-year cycles. The benchmark predictions in Figure 2c represent a best-case scenario in which ITN coverage is rapidly brought to scale, but a more realistic scenario would be that ITN coverage levels would be scaled-up over the five-year planning cycle. At the end of a scaling-up period, *Pf*PR would therefore remain higher than the benchmark (Supplementary Online Information).

## Caveats

The benchmarks illustrate how mathematical models can provide guidance about the likely outcome of scaling-up ITNs, but the predictions come with caveats. A monitoring and evaluation framework for assessing the performance of control programs based on parasitological markers will depend on the local entomological context for transmission, including vector bionomics, mode of action of the insecticides in the nets [14], observed levels of ITN ownership and use [4], the degree of heterogeneous biting, seasonal fluctuations in mosquito populations, changing weather, changes in malaria control, and changing socioeconomic status of countries. In particular, these predictions must be revised if national drug policies abandon failing drugs and adopt artemisinin combination therapies while simultaneously scaling-up ITNs: increased use of effective drugs also reduces transmission [37]. Analysis of steady states may not be useful in places with high inter-annual variability in transmission. Mathematical models can be adapted to reflect differences in the local ecology, provided that there is some additional information about the inputs. In practice, information about temporal trends and spatial variability in malaria transmission is usually not available. This analysis represents a starting point for planning that can be improved upon as more information about transmission in a specific context becomes available.

Taken together, baseline endemicity and uncertainty about heterogeneous biting, immunity, and vector bionomics suggest highly unpredictable endpoints after reaching universal coverage, as prescribed by RBM. Monitoring and evaluation across the transmission spectrum and across the range of dominant vector species should aim to establish context-specific expectations and goals.

## Conclusion

Mathematical models establish basic expectations about the changes in *Pf*PR as a function of ITN coverage. These can be used to establish rationally defined endpoints, timelines and criteria for monitoring and evaluation of ITN programs. A limitation for planning has been poor information about the global distribution of malaria risk, but a global map and an open-access database describing *Pf*PR have now been published, and these provide a basis for regional planning [29]. In practice, information about historical trends in other factors and spatial variability in malaria transmission is usually not available at scale. The models suggest that it is possible to transform malaria epidemiology across Africa in the short-to-medium-term by achieving high levels of ITN ownership use among all members of the population living across the diverse endemicity spectrum [29]. The timelines for a transition to low, stable endemic control is achievable over the next 5–10 years for much of the continent. More importantly, this impact can be predicted and measured. Ongoing surveillance, including parasitological monitoring, is imperative to evaluate the theory in the local context and update programmatic goals. Following adaptations to existing national sample survey methodologies promoted by RBM (Box 1), the international community can map progress and its contribution to the changing landscape of malaria in Africa [51].

## Figures and Tables

**Table 1:** Benchmark targets for ITN effective coverage, defined as ownership multiplied by the rate of use^a^

	*To halve Pf*PR			*To reach* 1% *Pf*PR		
*Pf*PR	*ϕ′* *=* *0%*	*ϕ′* *=* *10%*	*ϕ′* *=* *20%*	*ϕ′* *=* *0%*	*ϕ′* *=* *10%*	*ϕ′* *=* *20%*
5%	4%	14%	24%	7%	17%	27%
10%	8%	18%	28%	15%	25%	34%
15%	12%	22%	31%	23%	32%	41%
20%	16%	26%	35%	30%	39%	48%
25%	20%	29%	38%	37%	46%	54%
30%	24%	33%	42%	45%	52%	60%
35%	28%	37%	46%	51%	59%	65%
40%	33%	42%	50%	58%	65%	72%
45%	38%	46%	54%	65%	71%	76%
50%	42%	51%	59%	71%	77%	81%
55%	49%	56%	64%	77%	82%	86%
60%	55%	62%	69%	83%	87%	92%
65%	62%	69%	74%	89%	92%	96%
70%	70%	75%	80%	94%	98%	*
75%	78%	83%	87%	99%	*	*
80%	86%	90%	93%	*	*	*

a The first three columns give the ITN effective coverage target required to reduce *Pf*PR by 50% from the baseline. The next three columns report the ITN coverage required to reduce *Pf*PR to 1%. Each column represents a different ITN coverage at the baseline (*ϕ′*). The asterisk indicates *Pf*PR values for which a 1% *Pf*PR is not attainable with ITNs alone.

## Box 1 Malaria Indicators for monitoring and evaluation

Most malaria morbidity and mortality occurs in children under five years of age. Since the inception of Roll Back Malaria (RBM), national Malaria Indicator Surveys managed as Demographic and Health Surveys (DHS) or Multiple Indicator Surveys (MICS) have focused on coverage indicators among pregnant women or children under five years old. However, theory suggests that the best way to protect the most vulnerable people and to achieve stable endemic control will be to shift the emphasis from the most vulnerable and extend insecticide-treated bednets (ITN) coverage to the whole population [19]. Thus, monitoring ITN ownership and use must include all age groups in a community.

More recently, there has been a trend toward including infection prevalence in young children as part of national sample surveys, but the *Pf*PR in children under five years of age is difficult to interpret as a measure of transmission [27]. The standard *Pf*PR measured in children older than two years of age but younger than ten years of age has many advantages, including continuity with historical measures of malaria infection risk [20,52]. The correspondence with the steady state *Pf*PR makes it useful for applying epidemiological theory [27]. The *Pf*PR in 2–10-year-olds thus provides a reasonable index for planning, monitoring and evaluating progress. *Pf*PR changes sufficiently rapidly that it provides a measure of recent exposure, but sufficiently slowly that it provides a good average measure of exposure within the past 1–2 years. In general, reductions in *Pf*PR provide a good descriptor of *Pf*PR in the whole population and the parasite reservoir for ongoing transmission. National sample surveys should be encouraged to ensure a more efficient age sampling for parasite prevalence to track the impact of scaled intervention coverage.

## References

[1] Yamey G. (2004). Roll Back Malaria: a failing global health campaign. Br. Med. J..

[2] WHO (2005). Global Strategic Plan. Roll Back Malaria. 2005–2015.

[3] RBMP (2008). The Global Malaria Action Plan for a Malaria Free World. Roll Back Malaria Partnership.

[4] Noor A.M. (2009). Insecticide-treated net coverage in Africa: mapping progress in 2000-07. Lancet.

[5] Fegan G.W. (2007). Effect of expanded insecticide-treated bednet coverage on child survival in rural Kenya: a longitudinal study. Lancet.

[6] Barnes K.I. (2005). Effect of artemether-lumefantrine policy and improved vector control on malaria burden in KwaZulu-Natal, South Africa. PLoS Med..

[7] Bhattarai A. (2007). Impact of artemisinin-based combination therapy and insecticide-treated nets on malaria burden in Zanzibar. PLoS Med..

[8] Grabowsky M. (2008). The billion-dollar malaria moment. Nature.

[9] Nyarango P.M. (2006). A steep decline of malaria morbidity and mortality trends in Eritrea between 2000 and 2004: the effect of combination of control methods. Malar. J..

[10] Okiro E.A. (2007). The decline in paediatric malaria admissions on the coast of Kenya. Malar. J..

[11] Otten M. (2009). Initial evidence of reduction of malaria cases and deaths in Rwanda and Ethiopia due to rapid scale-up of malaria prevention and treatment. Malar. J..

[12] Teklehaimanot H.D. (2009). Malaria in Sao Tome and Principe: on the brink of elimination after three years of effective antimalarial measures. Am. J. Trop. Med. Hyg..

[13] Killeen G.F., Smith T.A. (2007). Exploring the contributions of bed nets, cattle, insecticides and excitorepellency to malaria control: a deterministic model of mosquito host-seeking behaviour and mortality. Trans. R. Soc. Trop. Med. Hyg..

[14] Le Menach A. (2007). An elaborated feeding cycle model for reductions in vectorial capacity of night-biting mosquitoes by insecticide-treated nets. Malar. J..

[15] Hill J. (2006). Insecticide-treated nets. Adv. Parasitol..

[16] Lengeler C. (2004). Insecticide-treated bed nets and curtains for preventing malaria. Cochrane Database Syst. Rev..

[17] Hawley W.A. (2003). Community-wide effects of permethrin-treated bed nets on child mortality and malaria morbidity in western Kenya. Am. J. Trop. Med. Hyg..

[18] Howard S.C. (2000). Evidence for a mass community effect of insecticide-treated bednets on the incidence of malaria on the Kenyan coast. Trans. R. Soc. Trop. Med. Hyg..

[19] Killeen G.F. (2007). Preventing childhood malaria in Africa by protecting adults from mosquitoes with insecticide-treated nets. PLoS Med..

[20] Hay S.I. (2008). Measuring malaria endemicity from intense to interrupted transmission. Lancet Infect. Dis..

[21] Macdonald G. (1956). Theory of the eradication of malaria. Bull WHO.

[22] Dietz K. (1993). The estimation of the basic reproduction number for infectious diseases. Stat. Meth. Med. Res..

[23] Macdonald G. (1957). The Epidemiology and Control of Malaria.

[24] Smith D.L. (2007). Revisiting the basic reproductive number for malaria and its implications for malaria control. PLoS Biol..

[25] Hay S.I. (2000). Annual *Plasmodium falciparum* entomological inoculation rates (EIR) across Africa: literature survey, internet access and review. Trans. R. Soc. Trop. Med. Hyg..

[26] Guerra C.A. (2007). Assembling a global database of malaria parasite prevalence for the Malaria Atlas Project. Malar. J..

[27] Smith D.L. (2007). Standardizing estimates of the *Plasmodium falciparum* parasite rate. Malar. J..

[28] Guerra C.A. (2008). The limits and intensity of *Plasmodium falciparum* transmission: implications for malaria control and elimination worldwide. PLoS Med..

[29] Hay S.I. (2009). World malaria map: *Plasmodium falciparum* endemicity in 2007. PLoS Med..

[30] Macdonald G., Göeckel G.W. (1964). The malaria parasite rate and interruption of transmission. Bull. WHO.

[31] Ross R. (1908). Report on the Prevention of Malaria in Mauritius.

[32] Ross R. (1911). The Prevention of Malaria.

[33] Dietz K. (1974). A malaria model tested in the African savannah. Bull. WHO.

[34] Dietz K., Wernsdorfer W., McGregor I. (1988). Mathematical models for transmission and control of malaria. Principles and Practice of Malaria.

[35] Dye C., Hasibeder G. (1986). Population dynamics of mosquito-borne disease: effects of flies which bite some people more frequently than others. Trans. R. Soc. Trop. Med. Hyg..

[36] Koella J.C. (1991). On the use of mathematical models of malaria transmission. Acta Trop..

[37] Okell L.C. (2008). Modelling the impact of artemisinin combination therapy and long-acting treatments on malaria transmission intensity. PLoS Med..

[38] McKenzie F.E., Bossert W.H. (2005). An integrated model of *Plasmodium falciparum* dynamics. J. Theor. Biol..

[39] Smith T. (2006). Mathematical modeling of the impact of malaria vaccines on the clinical epidemiology and natural history of *Plasmodium falciparum* malaria: overview. Am. J. Trop. Med. Hyg..

[40] Moskovsku S.D. (1967). A further contribution to the theory of malaria eradication. Bull. WHO.

[41] Smith D.L. (2005). The entomological inoculation rate and *Plasmodium falciparum* infection in African children. Nature.

[42] Beier J.C. (1999). Short report: entomologic inoculation rates and *Plasmodium falciparum* malaria prevalence in Africa. Am. J. Trop. Med. Hyg..

[43] Hay S.I. (2005). Urbanization, malaria transmission and disease burden in Africa. Nat. Rev. Microbiol..

[44] Garrett-Jones C. (1964). Prognosis for interruption of malaria transmission through assessment of the mosquito's vectorial capacity. Nature.

[45] Killeen G.F. (2000). A simplified model for predicting malaria entomologic inoculation rates based on entomologic and parasitologic parameters relevant to control. Am. J. Trop. Med. Hyg..

[46] Rowe A.K. (2006). The burden of malaria mortality among African children in the year 2000. Int. J. Epidemiol..

[47] Snow R.W. (1997). Relation between severe malaria morbidity in children and level of *Plasmodium falciparum* transmission in Africa. Lancet.

[48] Snow R.W., Marsh K. (2002). The consequences of reducing transmission of *Plasmodium falciparum* in Africa. Adv. Parasitol..

[49] Smith D.L., Hay S.I. (2009). Endemicity response timelines for *Plasmodium falciparum* elimination. Malar. J..

[50] Bailey N.T.J. (1982). The Biomathematics of Malaria.

[51] Greenwood B. (2008). Malaria: progress, perils, and prospects for eradication. J. Clin. Invest..

[52] Metselaar D., van Thiel P.H. (1959). Classification of malaria. Trop. Geogr. Med..

