# Supplementary material for: Predicting changing malaria risk after expanded insecticide-treated net coverage in Africa
Source: Trends Parasitol. 2009 Nov;25(11):511–6. doi: 10.1016/j.pt.2009.08.002 (PMC2768685; doi:10.1016/j.pt.2009.08.002)
Supplement: Supplementary file 1 [file mmc1.pdf]

Supplementary Information:
Predicting Malaria Control Endpoints and Timelines

David L. Smith, Simon I. Hay, Abdisalan M. Noor, Robert W. Snow

Changes in malaria endemicity under scaled-up ITN coverage in Africa can be predicted by combining a malaria transmission model and a control model that relates ITN coverage levels to a transmission effect size, defined here as a proportional reduction in the reproductive number: $R_0/R_C(\phi)$. A previously published ITN control model describes a non-linear relationship between ITN coverage and the effect size on transmission [1]. The malaria transmission model describes a non-linear relationship between the equilibrium *Pf*PR, *Pf*EIR, and PfR_0[2,3]. An analogous queuing model describes the timelines [4]. These models are described again here.

ITN control Model The ITN effect size on transmission follows the model previously published by Le Menac'h *et al.*[1]. The feeding-cycle model for ITNs assumes that ϕ represents the proportion of encounters between humans and mosquitoes that are altered by the presence of a net. This is called effective coverage, and it is usefully approximated by ownership multiplied by the frequency of use. The model predicts changes in vectorial capacity, which is directly proportional to R_0 or $R_C(\phi)$. The effect size, $R_0/R_C(\phi)$ is a function of effective coverage, ϕ, which is illustrated graphically in Figure 1b.

The reductions in vectorial capacity depend on two parameters that describe what happens when a mosquito encounters a human protected by a net, the fraction that end in a mosquito being killed (δ) or repelled (ζ). The reductions in vectorial capacity also depend on the bionomics of the vector at the baseline, when there is no ITN coverage (denoted by the subscript 0), including the lifespan ($1/g_0$), the proportion of bites on humans (Q_0), the length of the feeding cycle (f_0), and the time to sporogony (n).

Given estimates of these six parameters, we can predict the effect size achieved by ITNs as they scale up over time, $\phi(t)$. Four species have been characterized [1,5]. The benchmark results depend on one particular set of parameters and the parameters for one vector (see Table S1). That vector was chosen as the benchmark because it was an important African vector, and also because the effect size was approximately equal to the geometric mean response of two *An. gambiae* species from different places (Figure 1b). The assessment of uncertainty is based on the distributions in Table S1, and the associated variability in effect size is illustrated in Figure S1.

Malaria Transmission Model The following summarizes previously published descriptions and analysis of a malaria transmission model [2–4,6]. The notation and parameters are described in those publications, and tables of the terms and parameters are described in Box S1 and Box S2.

Steady States: The following model describes a population where *Pf*PR is stratified by exposure. *Pf*EIR (denoted $\mathcal{E}$ in equations) defines the population average exposure, but each population stratum has a distribution of biting weights, ω, such that the malaria exposure within a stratum is $\omega\mathcal{E}$. Clearance follows the assumption of Dietz, *et al.*[7]. The prevalence of malaria in a risk stratum is X_ω, and the dynamics are described by the equation:

$$\dot{X}_\omega = b\omega\mathcal{E}(1 - X_\omega) - \frac{b\omega\mathcal{E}}{e^{b\omega\mathcal{E}/r} - 1} X_\omega \tag{1}$$

It is assumed that ω is *Gamma* distributed with a mean of 1 and a variance α, so that exposure is also *Gamma* distributed with a mean $\mathcal{E}$ and a squared coefficient of variation of α. The population prevalence integrates over all risk strata:

$$X = \int_0^\infty \Gamma(\omega|\alpha) X_\omega d\omega. \tag{2}$$

At the steady state:

$$\bar{X} = 1 - \left(1 + \frac{b\mathcal{E}\alpha}{r}\right)^{-1/\alpha} \tag{3}$$

This equation fits the relationship between *Pf*EIR and *Pf*PR in children for $b/r \approx 0.45/yr$ and $\alpha \approx 4.2$ [8].

The dynamics of infections in mosquitoes generally follow the classical as-

sumptions [6], except that the rate of infection is modified to consider heterogeneous biting [3]. The probability that a mosquito becomes infected after biting a human is defined by the formula:

$$\tilde{X} = \int_0^\infty \omega c(\omega\mathcal{E})\Gamma(\omega|\alpha)d\omega \tag{4}$$

Following Smith, *et al.*[3]:

$$R_0 = \frac{bc_0}{r}\mathcal{E}\frac{S+\tilde{X}}{\tilde{X}}(1+\alpha) \tag{5}$$

If no estimate of $\mathcal{E}$ is available, then it can be inferred from $\bar{X}$ using Eq. 3:

$$\mathcal{E} = \frac{r\left(\left(1-\bar{X}\right)^{-\alpha}-1\right)}{b\alpha} \tag{6}$$

which gives a relationship between R_0 and $\bar{X}$:

$$R_0 = \frac{1+\alpha}{\alpha}\left(\left(1-\bar{X}\right)^{-\alpha}-1\right)c_0\left(1+\frac{S}{\tilde{X}}\right) \tag{7}$$

The formula is mainly a function of the *Pf*PR and the degree of heterogeneous biting (α). Other parameters that modify the expression are the baseline infectivity c_0, and the stability index (Box S2).

The benchmark approximation was made by assuming that that $c(\omega\mathcal{E}) = c_0$, so that:

$$\tilde{X} = c_0\left(1-\left(1-\bar{X}\right)^{(1+\alpha)/\alpha^2}\right) \tag{8}$$

This formula tends to underestimate R_0 if there is transmission blocking immunity. To estimate PfR_0 from *Pf*PR for some assumption about transmission blocking immunity, we use Eq. 6, along with Eq. 7 to get $\tilde{X}$ as a function of only $\bar{X}$, and the atomic parameters b/r (which always appear together) and $c_0 = 0.5$ and the index $S = 1$.

The functions can be used to describe changes in the steady state relationships under malaria control. Given a reduction in vectorial capacity as a function of ITN coverage, $V(\phi)$, we can predict the change in steady state under malaria

control, starting from a population without malaria control, using the formulas:

$$\bar{X}(\phi = 0) \overset{F}{\rightleftharpoons} R_0 \overset{V(\phi)}{\rightleftharpoons} R_C(\phi) \overset{F^{-1}}{\rightleftharpoons} \bar{X}(\phi) \tag{9}$$

The functions describing the algorithm for predicting the steady state *Pf*PR in the presence and absence of control ($\bar{X}(\phi)$ and $\bar{X}_0$, respectively) are given on top of the directional arrow. The functions can be inverted, and this also makes it possible to predict the changes in *Pf*PR at the steady state starting from one level of malaria control (ϕ') and improving (or relaxing) to another ϕ:

$$\bar{X}(\phi') \overset{F}{\rightleftharpoons} R_C(\phi') \overset{V^{-1}(\phi')}{\rightleftharpoons} R_0 \overset{V(\phi)}{\rightleftharpoons} R_C(\phi) \overset{F^{-1}}{\rightleftharpoons} \bar{X}(\phi) \tag{10}$$

R-code is freely available upon request, and a demonstration version is available at `http://www.map.ox.ac.uk`.

Timelines: To compute the timelines, a different set of equations is required; these have described in detail elsewhere, and are repeated here [4]. Minor differences between the steady state of these equations and of the equation above are introduced because the risk strata are subdivided into a finite number of compartments. The equations are analogous, in the sense that they make the same assumptions about the biology, and they have the same steady states in a limiting case on the mesh of compartments.

Let j subscripts denote a subpopulation with biting weight ω_j that comprises a fraction W_j of the whole population. Let $x_{m,j}$ denote the fraction of that subpopulation with a given MOI, m. Thus, $\sum_m x_{m,j} = 1$, for all j. The changes in the proportion uninfected within the jth population stratum is:

$$\begin{aligned} \dot{x}_{0,j} &= -h_0 x_{0,j} + r x_{1,j} \\ \dot{x}_{m,j} &= -(h_j + \rho_m) x_{m,j} + \rho_{m+1} x_{m+1} + h_j x_{m-1,j}; \end{aligned} \tag{11}$$

Here, we have taken $\rho_m = rm$, and $h_j = b\mathcal{E}$. The parasite rate is defined to be

$$X = \sum_j W_j (1 - x_{0,j}). \tag{12}$$

The probability that a mosquito becomes infected after biting a human, denoted

$\tilde{X}$ and called net infectivity, is given by the formula:

$$\tilde{X} = \sum_j c\omega_j W_j (1 - x_{0,j}). \tag{13}$$

The dynamic of infections in mosquitoes follow the same logic as above [3], such that *Pf*EIR is given by the equation:

$$\mathcal{E} = R_0 \frac{r\tilde{X}}{1 + s\tilde{X}} \tag{14}$$

The biting weights were given by a *Gamma*-like distribution, as described elsewhere [4]. Simulated timelines were produced by letting the equilibrium come to a specified equilibrium determined by the vectorial capacity, then instituting control. ITN coverage was simulated by changing the vectorial capacity, according to a model.

A. Sensitivity Analysis

Variability in the ITN effect size is realted to vector bionomics, as described elsewhere [1]. Another source of heterogeneity in the outcome the translation from *Pf*PR back to *Pf*EIR, which is largely ascribed to differences in the degree of biting heterogeneity. Taken together, baseline endemicity and uncertainty about heterogeneous biting, immunity, and vector bionomics suggest highly unpredictable endpoints after reaching universal coverage, as prescribed by RBM. Monitoring and evaluation across the transmission spectrum and across the range of dominant vector species should aim to establish context-specific expectations and goals. Figure 2b is based on the parameter values and distributions reported in Table S1, we have also generated a predicted effect size distribution (Figure S1).

The relationship between the *Pf*PR, the *Pf*EIR, and the PfR_0 is strongly affected by the degree of heterogeneous biting, described by α [4,9], and illustrated in Figure 1a. The fitted model describes $\alpha = 4.2$ as an average degree of biting heterogeneity, but the degree of biting heterogeneity can vary among populations, depending on context. Here, α is assumed to be drawn from a normal distribution with mean 4.2 and variance 1. Variability associated only with the distribution of α are illustrated in (Figure S2). Similarly, two populations with the same PfEIR

but different values of *Pf*PR, malaria would be more difficult to eliminate in the population with the lower *Pf*PR, because biting is more intense on a smaller fraction of the population.

Finally, because the relationship between ITN effective control and the ITN effect size is greater than log-linear, as illustrated in Figure 1b. This implies that if ITNs are scaled-up steadily over a period of time, then the greatest effects will not be realized until effective coverage is near the target maximum. This leads to different estimates of the *Pf*PR over time. To illustrate these differences, two tables were created by simulating a steady scaling up of ITN effective coverage over a five-year period (Tables S2 & S3).

With variability in vector bionomics and heterogeneous biting, the variable outcome is shown in Figure S3. Differences between the benchmark prediction and some actual outcome can also occur because of other reasons: Our analysis also assumes that there has been no major change in any other important factors, such as drought or contemporary changes in other modes of malaria control, such as a change in drug policy. If there has been a change, these must be taken into account when assessing the effects that are attributable to ITNs.

Parameter	1	2	3*	4	Sensitivity
$p_0 = e^{-g_0}$	0.83	0.9	0.94	0.86	TR(0.7,0.9, 0.95)
$t_0 = 1/f_0$	2.7 d	2 d	3 d	2.3 d	TR(2,3,5)
Q_0	0.95	0.9	0.75	0.72	TR(0.7,0.9,0.95)
n	10.7	11.6	10.3	9	TR(8,10,13)
δ	0.3				TR(0.05, 0.3, 0.5)
ζ	0.1				TR(0.05, 0.1, 0.3)
α	4.2				normal(4.2, 1)
c	0.5				0.5

Table S1 A table summarizing the uncertainty analysis. Four vectors with well-characterized bionomics (using data from Killeen *et al.*[5]) , are 1. *Anopheles gambiae* from Namawala; 2. *A. gambiae* from Kaduna; 3. *A. arabiensis* from Kankiya; and 4. *A. puntulatus* from Butelgut. *A. arabiensis* had approximately the geometric mean of the two *An. gambiae* vectors.

PfPR	ϕ at the end of five years																				
	0	0.05	0.10	0.15	0.20	0.25	0.30	0.35	0.40	0.45	0.50	0.55	0.60	0.65	0.70	0.75	0.80	0.85	0.90	0.95	1.00
	5%	4%	3%	2%	1%	1%	1%	1%	0%	0%	0%	0%	0%	0%	0%	0%	0%	0%	0%	0%	0%
	10%	8%	7%	6%	5%	4%	3%	2%	2%	1%	1%	1%	1%	1%	1%	1%	0%	0%	0%	0%	0%
	15%	13%	12%	10%	9%	7%	6%	5%	4%	3%	3%	2%	2%	2%	1%	1%	1%	1%	1%	1%	1%
	20%	18%	17%	15%	14%	12%	10%	9%	8%	7%	6%	5%	4%	4%	3%	3%	3%	2%	2%	2%	2%
	25%	23%	22%	20%	18%	17%	15%	14%	12%	11%	9%	8%	7%	6%	6%	5%	5%	5%	4%	4%	4%
	30%	28%	27%	25%	23%	22%	20%	18%	17%	15%	14%	12%	11%	10%	9%	8%	8%	7%	7%	7%	7%
	35%	33%	32%	30%	29%	27%	25%	23%	22%	20%	19%	17%	16%	14%	13%	12%	12%	11%	11%	10%	10%
	40%	39%	37%	35%	34%	32%	31%	29%	27%	25%	24%	22%	21%	19%	18%	17%	16%	15%	15%	15%	14%
	45%	44%	42%	41%	39%	38%	36%	34%	33%	31%	29%	28%	26%	25%	23%	22%	21%	20%	20%	19%	19%
	50%	49%	48%	46%	45%	43%	42%	40%	38%	37%	35%	34%	32%	30%	29%	28%	27%	26%	25%	25%	25%
	55%	54%	53%	52%	50%	49%	47%	46%	44%	43%	41%	40%	38%	37%	35%	34%	33%	32%	32%	31%	31%
	60%	59%	58%	57%	56%	55%	53%	52%	50%	49%	48%	46%	45%	43%	42%	41%	40%	39%	39%	38%	38%
	65%	64%	63%	62%	61%	60%	59%	58%	57%	55%	54%	53%	52%	50%	49%	48%	47%	46%	46%	45%	45%
	70%	70%	69%	68%	67%	66%	65%	64%	63%	62%	61%	60%	58%	57%	56%	55%	55%	54%	53%	53%	53%
	75%	75%	74%	73%	73%	72%	71%	70%	69%	68%	67%	66%	65%	64%	64%	63%	62%	62%	61%	61%	61%
	80%	80%	79%	79%	78%	77%	77%	76%	75%	75%	74%	73%	72%	72%	71%	70%	70%	69%	69%	69%	69%

Table S2 We simulated changes in PfPR for an ITN program that scaled-up ITN effective coverage from 0 to a specified target level at the end of five years. Each column represents a different target level of ITN coverage. Each row represents a different baseline PfPR in 5% increments. The entries in the table show the model predicted PfPR at the end of the five-year scaling-up period.

*Pf*PR	ϕ at the end of five years																				
	0.05	0.10	0.15	0.20	0.25	0.30	0.35	0.40	0.45	0.50	0.55	0.60	0.65	0.70	0.75	0.80	0.85	0.90	0.95	1.00	
5%	10.5	13.1	12.8	10.5	8.5	7.4	6.7	6.2	5.9	5.7	5.5	5.4	5.3	5.2	5.2	5.1	5	5	5	5	Years to Reach Endpoint
10%	7.1	9	11.4	13.4	13.3	11.4	9.4	8.2	7.5	7	6.6	6.4	6.2	6	6	5.9	5.8	5.8	5.8	5.8	
15%	6.1	7.2	8.2	9.8	12	13.6	13.3	11.2	9.4	8.3	7.7	7.2	6.9	6.7	6.6	6.5	6.4	6.3	6.2	6.2	
20%	5.8	6.4	7	7.7	8.5	9.9	12	13.8	13.4	11.3	9.5	8.5	7.9	7.5	7.2	7	6.9	6.8	6.7	6.7	
25%	5.6	6.2	6.6	7.1	7.6	8.2	9.2	10.8	13.1	13.9	12.4	10.2	8.9	8.2	7.8	7.5	7.3	7.2	7.1	7.1	
30%	5.5	6	6.4	6.7	7	7.4	7.8	8.5	9.5	11.3	13.6	13.8	11.5	9.6	8.7	8.2	7.8	7.6	7.5	7.4	
35%	5.4	5.9	6.3	6.6	6.8	7.1	7.4	7.8	8.3	9.1	10.5	13	14	12.1	9.9	8.9	8.3	8	7.8	7.7	
40%	5.4	5.9	6.2	6.5	6.7	6.9	7.1	7.4	7.7	8.1	8.7	9.6	11.5	13.9	13.2	10.5	9.2	8.5	8.2	8.1	
45%	5.3	5.8	6.1	6.4	6.6	6.8	7	7.2	7.5	7.7	8.1	8.5	9.4	11	13.8	13.4	10.5	9.2	8.6	8.3	
50%	5.3	5.8	6	6.3	6.5	6.7	6.9	7.1	7.3	7.6	7.8	8.1	8.5	9.2	10.7	13.6	13.4	10.4	9.2	8.7	
55%	5.2	5.6	6	6.2	6.4	6.6	6.8	7	7.2	7.4	7.6	7.8	8.1	8.4	9	10	12.8	13.8	10.5	9.3	
60%	5.2	5.6	5.9	6.1	6.4	6.5	6.7	6.9	7.1	7.3	7.5	7.7	7.9	8.2	8.5	9	10	13	13.3	10.1	
65%	5.1	5.5	5.8	6.1	6.2	6.5	6.6	6.8	7	7.2	7.3	7.6	7.8	8.1	8.3	8.7	9.2	10.1	13.5	12.1	
70%	5	5.4	5.8	6	6.2	6.4	6.5	6.7	6.9	7.1	7.2	7.5	7.7	7.9	8.2	8.4	8.8	9.4	10.4	14.3	
75%	4.9	5.4	5.6	5.9	6.1	6.3	6.5	6.6	6.8	7	7.2	7.3	7.6	7.8	8.1	8.3	8.7	9.1	9.7	11.4	
80%	4.8	5.3	5.5	5.8	6	6.1	6.4	6.5	6.7	6.8	7.1	7.2	7.5	7.7	7.9	8.2	8.5	8.9	9.4	10.1	

Table S3 We simulated changes in PfPR for an ITN program that scaled-up ITN effective coverage from 0 to a specified target level at the end of five years. We extended the simulations so that after five years, the maximum level of ITN effective coverage was sustained indefinitely. Each column represents a different target level of ITN coverage, and each row represents a different baseline PfPR in 5% increments. The numbers report the number of years elapsed, counting from the start of the program, before PfPR is within 1% of its endpoint.

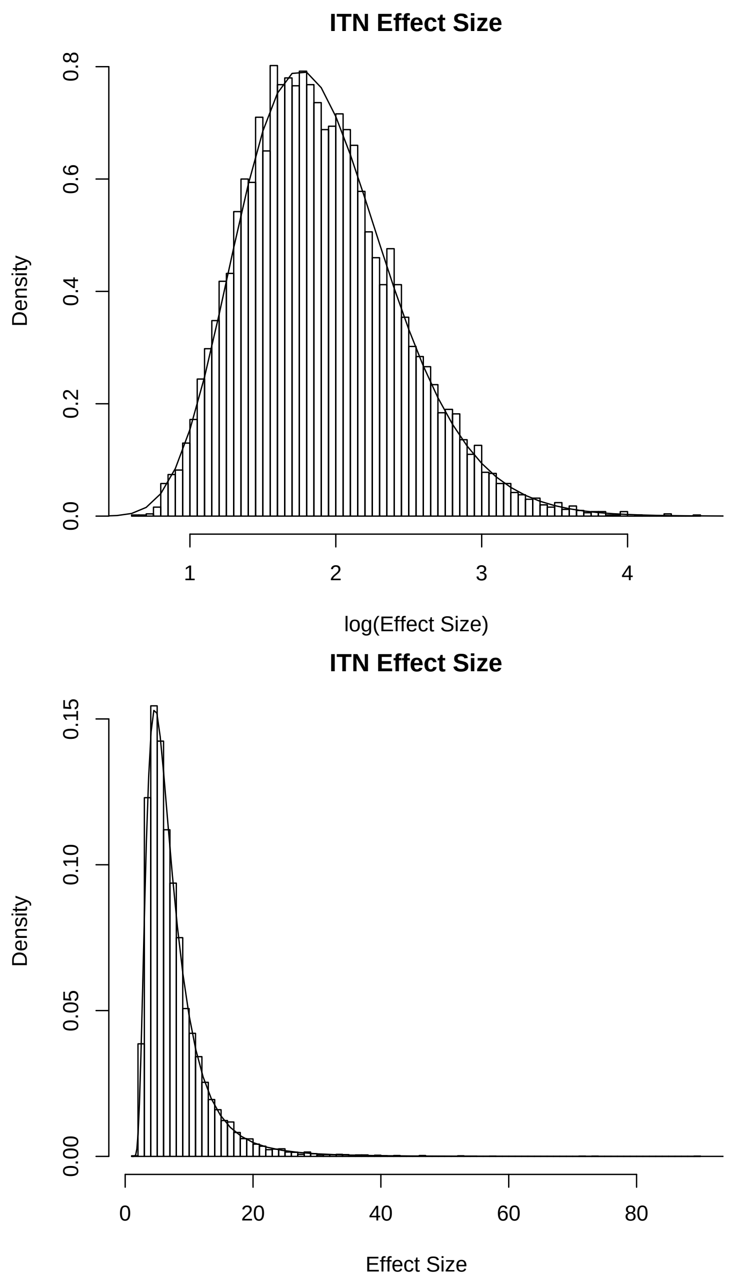

Figure S1 The variability in the predicted effect size achieved with 60% effective coverage, or 80% coverage and 75% use. These represent 10,000 monte carlo sample from the distributions reported in Table S1, the log effect size is Gamma distributed with shape parameter, $a \approx 13.4$ and scale paramater $s \approx 0.14$.

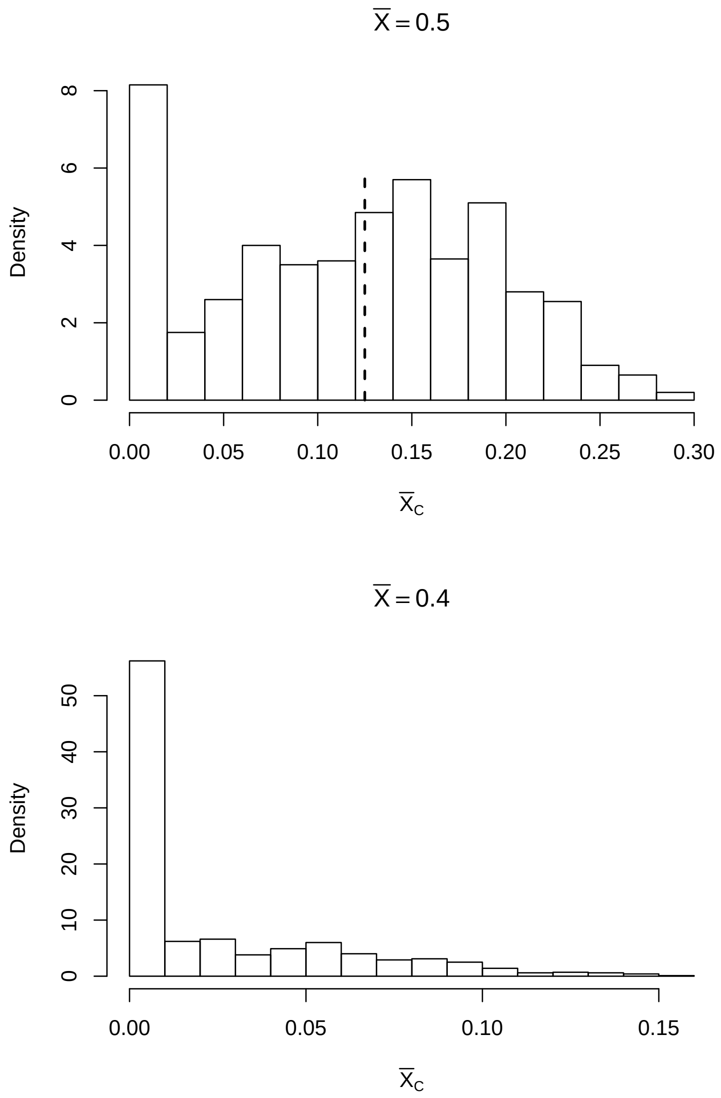

Figure S2 The variability in outcomes achieved when considering only variability in heterogeneous biting, starting from the reported initial endemicity value (i.e. $\bar{X}$ of 50% or 40%).

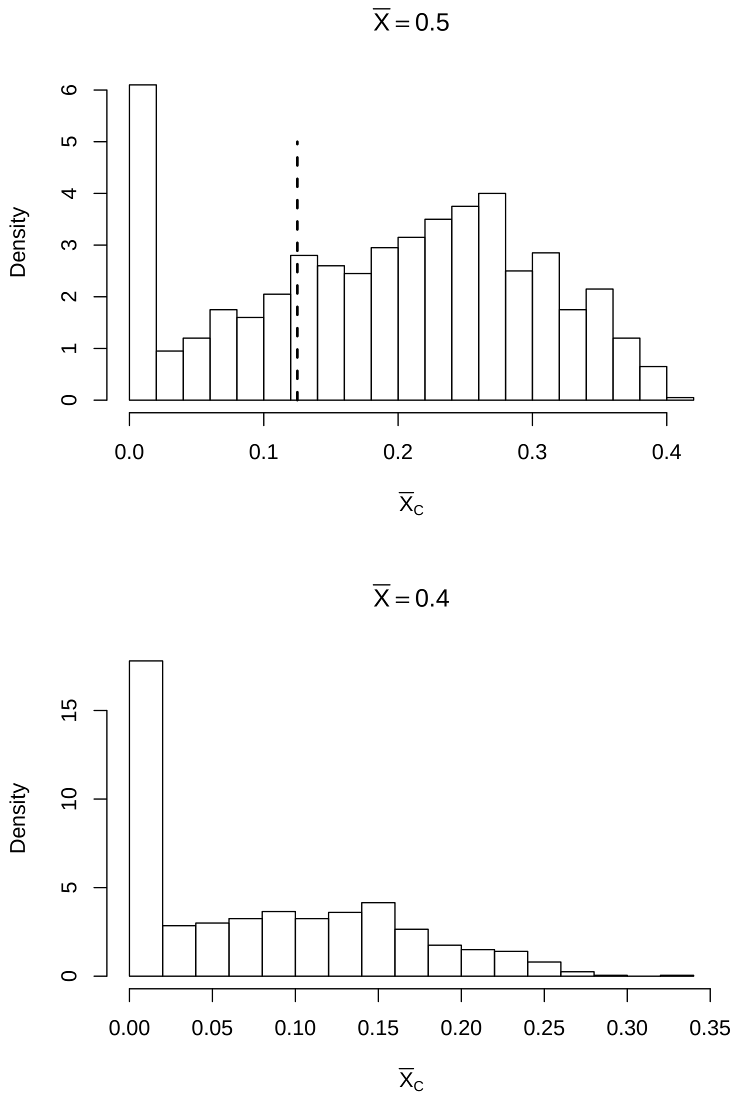

Figure S3

The variability in outcomes achieved when considering both the variability in heteroeneous biting and effect size, using the distributions reported in Table S1.

Box S1: Parameters and Terms

- λ: Recruitment of adult vectors, per human, per day
- g^{-1}: Average lifespan of an adult vector. The probability of surviving one day is $p = e^{-g}$.
- f^{-1}: Average duration of the vector feeding cycle
- Q: Human bloodmeals per bloodmeal
- n: Number of days to complete sporogony
- c_0: Efficiency of transmission to mosquitoes, measured at low endemicity.
- b: Efficiency of transmission to humans, measured at low endemicity. Here, we take $b = 0.8$.
- r: The waiting time to clear a simple, untreated infection, approximately 200 days.
- $\sqrt{\alpha}$: Coefficient of variation of human exposure. We take $\alpha^2 = 4.2$, the published best-fit estimate[8]. In the numerical simulations, $\alpha = \sum_j W_j(1-\omega_j)^2$.

Box S2: Indices of Transmission Intensity

- s: The stability index (fQ/g)
- h: The force of infection. In these models $h = b\mathcal{E}$.
- $\tilde{X}$: Net human infectiousness, the probability a mosquito becomes infected after biting a human. Here, we use $\tilde{X} = \int_0^\infty \omega c(\omega\mathcal{E})\Gamma(\omega|\alpha)d\omega$ for steady states and $\tilde{X} = \sum_j \omega_j W_j (1 - X_{0,j})$ for timelines.
- V: Vectorial capacity. $\lambda s^2 e^{-gn} = ma^2 e^{-gn}/g$.
- $\mathcal{E}$: Entomological inoculation rate, $\mathcal{E} = V\tilde{X}/(1 + s\tilde{X})$.
- $\bar{X}$: Standard *Pf*PR at the steady state
- R_0: The basic reproductive number, $bcV(1 + \alpha)/r$.

REFERENCES

1. Le Menach, A, Takala, S, McKenzie, F. E, Perisse, A, Harris, A, Flahault, A, & Smith, D. L. (2007) *Malar J* **6**, 10.

2. Smith, D. L, Dushoff, J, & McKenzie, F. E. (2004) *PLoS Biol* **2**, e368.

3. Smith, D. L, McKenzie, F. E, Snow, R. W, & Hay, S. I. (2007) *PLoS Biol* **5**, e42.

4. Smith, D. L & Hay, S. I. (2009) *Malar J* **8**, 87.

5. Killeen, G. F, McKenzie, F. E, Foy, B. D, Schieffelin, C, Billingsley, P. F, & Beier, J. C. (2000) *Am J Trop Med Hyg* **62**, 535–544.

6. Smith, D. L & McKenzie, F. E. (2004) *Malar J* **3**, 13.

7. Dietz, K, Molineaux, L, & Thomas, A. (1974) *Bull. Wld. Hlth. Org.* **50**, 347–357.

8. Smith, D. L, Dushoff, J, Snow, R. W, & Hay, S. I. (2005) *Nature* **438**, 492–495.

9. Dietz, K. (1988) in *Principles and Practice of Malaria*, eds. Wernsdorfer, W & McGregor, I. (Churchill Livingstone, Edinburgh, UK), pp. 1091–1133.
